# The cost-effectiveness of tafenoquine following screening with STANDARD™ G6PD screening for the treatment of vivax malaria in the Brazilian Public Health System

**DOI:** 10.1016/j.lana.2025.101216

**Published:** 2025-09-02

**Authors:** Henry Maia Peixoto, Luiza Lena Bastos Gottin, Jose Diego de Brito-Sousa, Vanderson Sampaio, Penny Grewal Daumerie, Elodie Jambert, Wuelton Monteiro, Marcus V.G. Lacerda, Angela Devine

**Affiliations:** a Faculdade de Medicina, Núcleo de Medicina Tropical, Universidade de Brasília, Brasília, Brazil; b Global Health Strategies, Rio de Janeiro, Brazil; c Fundação de Medicina Tropical Dr Heitor Vieira Dourado, Manaus, Brazil; d Escola Superior de Ciências da Saúde, Universidade do Estado do Amazonas, Manaus, Brazil; e Fundacão de Vigilância em Saude Rosemary Costa Pinto–FVS-RCP, Manaus, Brazil; f Medicines for Malaria Ventures, Geneva, Switzerland; g Escola Superior de Ciências da Saúde, Universidade do Estado do Amazonas, Manaus, Brazil; h Global and Tropical Health Division, Menzies School of Health Research, Charles Darwin University, Darwin, Northern Territory, Australia; i Centre for Health Policy, Melbourne School of Population and Global Health, The University of Melbourne, Melbourne, Victoria, Australia

**Keywords:** Vivax malaria, G6PD screening, Cost-effectiveness analysis, Tafenoquine

## Abstract

**Background:**

Vivax malaria requires radical cure to clear both the blood-stage and liver-stage parasites. Brazil, like most endemic countries, has been prescribing a 7-day primaquine regimen for radical cure without testing for glucose-6-phosphate-dehydrogenase (G6PD) deficiency to exclude those at risk of primaquine-induced haemolysis. Tafenoquine, a new single-dose drug for radical cure requires G6PD screening before prescription to ensure safety. This study aims to assess the cost-effectiveness of prescribing tafenoquine after semi-quantitative G6PD screening from the Brazilian Public Health System perspective.

**Methods:**

A decision tree model was developed for adults presenting with vivax malaria over 12-months. The *tafenoquine strategy* of semi-quantitative G6PD testing before prescription of single-dose tafenoquine to those with ≥70% G6PD activity was compared with: (1) *current practice*: 7-day low-dose primaquine (0.5 mg/kg/day) without G6PD screening and (2) *primaquine screening strategy:* 7-day low-dose primaquine (0.5 mg/kg/day) for patients with ≥30% G6PD activity determined by semi-quantitative G6PD screening. The primary outcome was the cost per disability-adjusted life-year (DALY) averted, compared with the Brazilian willingness-to-pay threshold of US$7752 (R$40,000).

**Findings:**

The *tafenoquine strategy* was US$2894 (R$14,934) per DALY averted compared to *current practice*, well below the willingness-to-pay threshold. The *tafenoquine strategy* dominated the *primaquine screening strategy*, averting 0.14 DALYs with cost savings of US$13 (R$66). In both comparisons, the *tafenoquine strategy* had a >98% likelihood of being cost-effective.

**Interpretation:**

The prescription of tafenoquine to those who test G6PD normal with a semi-quantitative test is a cost-effective strategy for the radical cure of vivax malaria in Brazil. While the cost-effectiveness in other settings may vary due to differences in costs and the epidemiology of vivax malaria and G6PD deficiency, the robustness of these findings should be reassuring, particularly where healthcare facilities expect to see a large number of patients annually.

**Funding:**

10.13039/501100004167Medicines for Malaria Ventures.

**Research in context**

**Evidence before this study**

Using the search terms (“g6pd” OR “Glucose-6-phosphate dehydrogenase”) AND “tafenoquine” AND (“cost-effectiveness” OR “cost”) AND “vivax”, we searched PubMed for studies reporting the cost-effectiveness of prescribing tafenoquine to those with >70% G6PD activity with quantitative screening. Only one study was identified. In this study from Brazil, a dynamic-transmission economic evaluation model was used to evaluate the cost-effectiveness of tafenoquine after G6PD screening as compared to primaquine without G6PD screening with incremental cost-effectiveness ratios of US$154–1836 per disability-adjusted life-year averted. Despite the initial investment cost for G6PD analysers, this study found a high likelihood of tafenoquine being cost-effective when the transmission is included, particularly in settings with low primaquine adherence and where pediatric formulations of tafenoquine are available.

**Added value of this study**

To capture the effect of tafenoquine on recurrences, a decision tree model was developed to estimate the costs and outcomes in terms of disability-adjusted life-years (DALYs) for adult vivax malaria patients over a 12-month time horizon from the Brazilian public healthcare system perspective. Compared to primaquine without G6PD screening, the ICER was US$2894 (R$14,934) per DALY averted, which was below the cost-effectiveness threshold of US$7752 (R$40,000). Tafenoquine dominated primaquine with G6PD screening, indicating that tafenoquine averted DALYs while saving costs. Both comparisons indicate that tafenoquine after G6PD screening is cost-effective.

**Implications of all the available evidence**

This study showed a high likelihood that prescribing tafenoquine to those with normal G6PD will be cost-effective in Brazil even when the results are limited to individual patients (e.g., the impact on onward transmission was not included). The results of this study led to the implementation of semi-quantitative G6PD testing and tafenoquine in the Brazilian Public Health System; this is the first malaria-endemic country to implement it. The high likelihood of cost-effectiveness lends credibility to the sustainability of investing in G6PD screening to ensure patient access to tafenoquine, which will be valuable for other countries in the region that are considering implementation.

## Introduction

The majority of malaria in Brazil is due to *Plasmodium vivax*, with an estimated 115,785 indigenous *P. vivax* cases reported in 2023 as compared to 21,491 *Plasmodium falciparum* cases.^1^ The control of vivax malaria is particularly challenging due to the dormant liver parasites (hypnozoites) that can cause multiple relapses after an initial infectious mosquito bite. The radical cure of *P. vivax* will be vital to successful malaria elimination efforts, ensuring that the liver-stage parasites are cleared in addition to the blood-stage treatment. Currently, only primaquine is widely used for radical cure; however, two recent clinical trials highlighted that tafenoquine was non-inferior to low-dose (0.5 mg/kg for 7 days) primaquine and can be administered as a single dose.^2^^,^^3^ The ability to administer tafenoquine in a single dose overcomes the significant challenges of getting patients to adhere to primaquine regimens that continue after malaria symptoms resolve.^4^, ^5^, ^6^, ^7^, ^8^, ^9^

Both primaquine and tafenoquine can lead to hemolysis in individuals with glucose-6-phosphate-hydrogenase (G6PD) deficiency, an inherited enzymopathy. Until recently, Brazil's policy and practice was to prescribe primaquine over seven days without screening for G6PD deficiency. While the more extended regimen enables patients to stop treatment if an adverse reaction occurs, hospitalization and mortality due to primaquine-induced hemolysis have been reported in patients with G6PD deficiency in Brazil.^10^, ^11^, ^12^ Tafenoquine requires a semi-quantitative G6PD test before the prescription of tafenoquine (300 mg to adults) to those testing G6PD normal. The semi-quantitative test also enables 7-day primaquine prescription (0.5 mg/kg daily) to those testing G6PD intermediate, while those testing G6PD deficient receive 0.75 mg/kg weekly for 8 weeks (PQ8W). The operational effectiveness of this treatment algorithm in Brazil is superior for preventing *P. vivax* recurrences.^13^

For new drugs to be included in the Brazilian Public Health System (*Sistema Único de Saúde* [SUS]), a submission had to be made to its Health Technology Assessment body, Brazilian National Commission for the Incorporation of Technology in the SUS (*Comissão Nacional de Incorporação de Tecnologias no Sistema Único de Saúde* [CONITEC]). This analysis describes the cost-effectiveness analyses completed for that submission,^14^ which was approved on the 5th of June, 2023, by CONITEC for the incorporation of TQ and G6PD testing in the SUS. The first comparison is tafenoquine after semi-quantitative G6PD screening to current practice in Brazil, which is a 7-day primaquine without G6PD screening. Since malaria guidelines in Brazil had been updated to require G6PD screening before primaquine at health facilities that have the capacity to perform G6PD tests, a second comparison evaluated tafenoquine following semi-quantitative G6PD screening compared to primaquine following semi-quantitative G6PD screening.

## Methods

### Model structure

A cost-effectiveness analysis over a 12-month time horizon using the SUS perspective in Brazil was conducted with a decision tree model for individuals presenting with *P. vivax* malaria in Brazil. The structure was adapted from a previously published model using R statistical software.^15^ The model structure was created using DARE software (Supplementary Figures S1 and S2).^16^ Since a pediatric version of tafenoquine is not yet available in Brazil, the model was designed for individuals over the age of 16 years presenting with uncomplicated vivax malaria. A cohort of 55 patients was assumed based on the mean number of patients per facility per year where semi-quantitative G6PD test devices would be placed.^17^

Different pathways are presented for males and females to account for their differences in risks and outcomes. Pregnant females should not be prescribed radical cure due to the unknown G6PD status of the fetus. Prior to the initiation of treatment for malaria, routine pregnancy testing is not done within SUS, so it was assumed that women would know whether they were pregnant. The cost of a pregnancy test was not included. Since G6PD is an X-linked disorder, males who have deficiency are hemizygous while females can be either homozygous or heterozygous with a range of G6PD expression levels. Accordingly, G6PD deficiency was divided into two groups: severe (<30% enzyme activity) and intermediate (30–70% enzyme activity). Lactating women breastfeeding children older than one month in Brazil are allowed to be treated with primaquine but not with tafenoquine.^18^

The following treatment strategy was explored:

#### Tafenoquine strategy

Semi-quantitative G6PD test and prescription of single-dose tafenoquine (300 mg) to those with ≥70% G6PD activity. Patients with intermediate test results (30–70% G6PD activity) are prescribed 7-day unsupervised primaquine therapy (0.5 mg/kg/day). Patients with <30% G6PD activity are prescribed PQ8W (0.75 mg/kg/dose).

The *tafenoquine strategy* was compared with the following two strategies in separate analyses:

#### Current practice

7-day unsupervised low-dose primaquine therapy (0.5 mg/kg/day) without G6PD screening.

#### Primaquine screening strategy

7-day unsupervised low-dose primaquine therapy (0.5 mg/kg/day) for patients with ≥30% G6PD activity determined by semi-quantitative G6PD screening. Patients with <30% G6PD activity are prescribed PQ8W (0.75 mg/kg/dose).

The CHEERS guidelines were used to ensure that the standards for economic evaluation reporting were met (Supplemental Material).

### The impact of radical cure

The effectiveness of radical cure was calculated using the efficacy of radical cure from a clinical trial evaluating the risk of recurrence over six months in patients treated with chloroquine alone, compared to patients treated with chloroquine plus 14 days of supervised primaquine for each *P. vivax* episode (0.25 mg/kg/day) compared to those treated with tafenoquine (300 mg) using data only from Brazil.^2^ The 14-day regimen is the same total dose of primaquine as Brazil uses over seven days, so the two regimens have the same efficacy. In total, 77% of those treated with chloroquine alone had at least one recurrence, and the relative risk following radical cure was 0.55 (Table 1). Using the formula from White,^20^ the fraction experiencing *n* relapses is *x*^*n*^ where *x* is the fraction of patients experiencing at least one relapse, and *n* is applied for each of eight cycles of 1.5 months. For those experiencing at least one recurrence, the mean number of recurrences was 3.8 without radical cure and 1.7 with radical cure. It was assumed that the efficacy of tafenoquine, low-dose primaquine, and PQ8W were equivalent.

**Table 1:** Model probability parameters and sources.

Parameter	Base case (low—high)	Distribution	Sources and notes
Annual number of vivax malaria patients per clinic	55 (1–2385)	Normal	^17^
Discount rate for the semi-quantitative G6PD device	0.05 (0–0.10)	Not varied in PSA	Ministry of Health
Lifetime of the semi-quantitative G6PD device, years	5 (3–10)	Normal	Assumption
Proportion of patients adhering to 7-day primaquine regimen	0.67 (0.54–0.84)	Beta	for^5^ base case, for^6^ low value, meta-analysis of^7^, ^8^, ^9^ for high value
Proportion of vivax malaria patients who are male	0.61 ± 20%	Beta	^19^
Proportion who have at least one recurrence in the following year if treated with chloroquine alone	0.77 (0.40–0.85)	Beta	^2^
Relative risk of having at least one recurrence in the following year if treated with chloroquine + radical cure compared to chloroquine alone	0.55 (0.39–0.78)	Lognormal	^2^
Mean number of recurrences in those treated with chloroquine alone who have at least 1 recurrence	3.8 ± 30%	Normal	^2^^,^^20^
Mean number of recurrences in those treated with chloroquine + radical cure who have at least 1 recurrence	1.7 ± 30%	Normal	^2^^,^^20^
Proportion of males with severe G6PD deficiency (<30%)	0.056 ± 20%	Beta	^21^
Proportion of females with severe G6PD deficiency (<30%)	0.003 ± 20%	Exponential	Applied Heidy Weinberg to the proportion of males with severe G6PD deficiency.^21^
Proportion of females with intermediate G6PD activity (30–70%)	0.106 ± 20%	Beta	Applied Heidy Weinberg to the proportion of males with severe G6PD deficiency.^21^
Sensitivity of semi-quantitative G6PD test for severe deficiency in males	0.999 (0.94–1.00)	Beta	While the study found 100% sensitivity, this has been lowered to 99.9% to allow for some human error.^22^
Proportion of true intermediates that test as <30% activity (no radical cure)	0.49 ± 20%	Beta	^22^
Proportion of true intermediates that test as ≥70% (prescribed TQ)	0.06 ± 50%	Beta	^22^
Specificity of semi-quantitative G6PD test	0.95 (0.90–1.00)	Beta	^22^
Probability of hemolysis requiring hospitalization if G6PDd (<30% activity) and prescribed radical cure	0.038 (0.015–0.061)	Beta	Base case is midpoint between low and high values^11^.
Probability of hemolysis if G6PD intermediate (30–70% activity) and prescribed radical cure	0.031 (0.001–0.038)	Exponential	^23^
Mortality due to hemolysis if G6PD deficient and prescribed radical cure	0.011 (0.005–0.016)	Beta	^11^
Proportion of females who are pregnant	0.023 ± 20%	Beta	^24^
Proportion of females who are lactating	0.06 ± 20%	Beta	^25^

PSA, probabilistic sensitivity analysis.

Primaquine efficacy is dependent upon the total dose received. Since primaquine therapy in the trial was supervised, the efficacy represents complete adherence to the regimen. A study of adherence to a 7-day regimen in Brazil showed that only 67% of patients took all of their primaquine doses.^5^ The effectiveness of primaquine was assumed to be binary: either a patient took the full regimen and had recurrences associated with a full course, or they did not take the full regimen and had the recurrences associated with not receiving radical cure. Accordingly, the effectiveness of unsupervised primaquine was derived from a mixed result of patients who adhered to their regimen and those who did not:

$$MR=propR*RR*\;{NR}_{rc}*propA\;+\;propR*{NR}_{cq}\;*\left(1-propA\right)$$

where MR is the mixed result, *propR* is the proportion having at least one recurrence if receiving chloroquine without radical cure, *RR* is the relative risk of having at least one recurrence if receiving low-dose primaquine as compared to no radical cure, ${NR}_{rc}$ is the expected relapses with radical cure, and ${NR}_{cq}$ is the expected relapses without radical cure and propA is the proportion adherent to their primaquine regimen. For each recurrence, the cost and DALY value used were taken from clinical episodes, severe malaria episodes and episodes resulting in death which were weighted proportionally. The probability for severe *P. vivax* was derived from a meta-analysis of clinical studies using those with severe anemia; other symptoms due to severe *P. vivax* were not included.^26^ Supplementary Table S1 shows the impact of strategies on individuals based on their G6PD status and test result.

### G6PD deficiency and hemolytic risk

Data on G6PD status in individuals with vivax malaria in Brazil was unavailable; hence, the population prevalence of G6PD deficiency was used. Since G6PD activity may rise in patients with malaria,^27^ this assumption may overestimate the prevalence of G6PD deficiency. The SD Biosensor Standard G6PD test can accurately identify patients across a range of G6PD activity levels, and a 70% cut-off was applied before tafenoquine prescription in line with the approved label. The base case analysis assumed that all males with G6PD deficiency would have <30% G6PD activity. In a scenario analysis, this assumption was replaced by the assumption that 28% of males had intermediate G6PD activity using data from the TRuST study to inform that estimate.^25^

The probability of having a severe hemolytic episode in patients with severe G6PD deficiency treated with radical cure was 3.8%.^11^ It was assumed that 3.1% of individuals with intermediate G6PD deficiency who were prescribed radical cure (excluding PQ8W) would have a severe hemolytic episode requiring hospitalization.^23^ Of those requiring hospitalization, 1.1% were assumed to die as a result of hemolysis.^11^

### Costs

Where possible, the costs were obtained in Brazilian currency (Brazilian Real, R$) for the year 2020. Costs from other years were inflated.^28^ Costs were converted into US dollars (US$) using the average official exchange rate for 2020 (R$5.16 per US$).^29^ The main results are presented in both currencies. Table 2 shows the cost parameters. Costs of commodities and service delivery were taken from the Amazonas region of Brazil. The cost of G6PD screening with a semi-quantitative test included the device cost, an additional blood draw, a test strip, high and low-quality assurance (administered monthly), and training costs. Training costs for the semi-quantitative test included room rental, staff time, stationery, and catering. This came to R$298 per facility (R$149 per person with two people trained per facility). The training costs were divided by the number of patients per healthcare facility. The cost of hospitalization was included for severe hemolytic episodes for patients, including the cost of transfusion for those who required one. Recurrence costs include diagnostic tests, medications, and clinical costs for the number of recurrences (weighted by the proportion of severe cases). For the *tafenoquine strategy* and *primaquine screening strategy*, it was assumed that screening for G6PD deficiency would be needed for each recurrence.

**Table 2:** Model cost parameters and sources.

Parameter	Base case in US$ (low—high)	Distribution	R$, sources and notes
Hemolytic episode requiring hospitalization	87 (49–124)	Gamma	R$445.97 (254.26–637.38).^17^
Severe *P. vivax* recurrence	59 (51–66)	Gamma	R$302.63 (264.97–340.28).^17^
Primaquine treatment (full low-dose regimen)	0.43	Not varied in sensitivity analyses	R$2.2. Ministry of Health
Primaquine treatment (8-weekly)	0.74	Not varied in sensitivity analyses	R$3.8. Ministry of Health
Tafenoquine treatment	1.78 (1.42–2.09)	Gamma	R$9.2 (7.3–10.8). Assumption from Câmara de Regulação do Mercado de Medicamentos (CMED)
Initial episode and clinical *P. vivax* recurrence		Gamma	R$37 (19–56).^30^
Semi-quantitative G6PD test analyer	619 (375–688)	Gamma	R$3158 (1913–3509). Assumption from test distributor
Semi-quantitative G6PD test strip	6.8 (4.1–8.2)	Gamma	R$34 (21–42). Assumption from test distributor
Quality assurance for semi-quantitative test	20 (17–23)	Gamma	R$32 (19–50). Assumption from test distributor
Healthcare facility trained per year	58 ± 50%	Gamma	R$298 ± 50%.^13^ Assume 2 healthcare workers per facility; divided by the number of patients per year.
Blood draw	0.64 ± 50%	Gamma	R$3.3 ± 50%.^31^ Includes staff time from blood draw to G6PD result, lancet, gloves, cotton wool, sharps container & alcohol.

All costs are listed in 2020 United States dollars with the corresponding Brazilian Real (R$) in the notes.

### DALYs

Disability-adjusted life-years (DALYs) were used as the effectiveness measure. The DALY weights were taken from the 2017 Global Burden of Disease Study for malaria and anemia.^32^ These weights were combined with assumptions about the length of illness^15^ and life tables for Brazil for mortality.^33^ The DALY burden for each strategy included the initial episode and any severe hemolytic events associated with its treatment. Recurrences weighted by disease severity (clinical or severe) and mortality are also included. Table 3 shows the parameters used in the DALY calculations.

**Table 3:** Model parameters for the disability-adjusted life-years and sources.

Parameter	Base case (low, high)	Distribution	Sources and notes
Hospitalization due to severe *P. vivax* (for recurrences)	0.03 ± 50%	Beta	^10^
Mortality due to *P. vivax* (for recurrences)	0.0003 (0–0.0005)	Beta	with assumed range^10^
Length of disability for clinical malaria, days	3 (1–7)	Beta	Assumption
Length of disability for severe malaria, days	7 (3–10)	Beta	Assumption
Length of disability for anemia due to clinical malaria, months	1 (0.5–2)	Beta	Assumption
Length of disability for anemia due to severe malaria, months	3 (1–6)	Beta	Assumption
Disability weight for clinical malaria	0.051 (0.032–0.074)	Gamma	^32^
Disability weight for severe malaria	0.133 (0.088–0.190)	Gamma	^32^
Disability weight for moderate anemia due to vivax malaria	0.052 (0.034–0.076)	Gamma	^32^
Disability weight for severe anemia due to severe malaria or hemolysis	0.149 (0.101–0.209)	Gamma	^32^
Life expectancy for males aged 25–29 in Brazil	50 ± 20%	Gamma	This was 18 after 5% discounting was applied^33^
Life expectancy for females aged 15–19 in Brazil	65 ± 20%	Gamma	This was 19 after 5% discounting was applied^33^

### Analyses

The total costs and DALYs will be calculated for each strategy. For both comparisons, the incremental cost-effectiveness ratios (ICER) was calculated:

$$ICER=\frac{{Cost}_{s\;}-\;{Cost}_{b}}{{DALY}_{b}\;-\;{DALY}_{s}}$$

where *Cost* is the total cost of the strategy and *DALYs* is the total DALYs of the corresponding strategy. In accordance with CONITEC Guidelines, a willingness to pay threshold of US$7752 (R$40,000) was used.^34^ This threshold can be considered conservative, particularly in light of CONITEC's observation that an alternative threshold of up to three times this value may be adopted for endemic diseases affecting low-income populations with limited therapeutic alternatives available.

A one-way sensitivity analysis was conducted to examine the impact of parameter values on the overall outcome. When available, low and high values were taken from 95% confidence intervals (CIs). When unavailable, the point estimate was varied by 20% or more to reflect the uncertainty (Table 1, Table 2, Table 3). The 10 parameters with the most significant impact on the results were reported.

A probabilistic sensitivity analysis (PSA) was conducted to incorporate the uncertainty of all parameters over 1000 sampling iterations using the parameter ranges used in the one-way sensitivity analysis. Table 2 lists the distributions used in the PSA. The sum of squared differences was minimized from the specified ranges to produce the shape values for the beta and gamma distributions and random numbers were generated from these distributions. The PSA produced a mean estimate and 95% credible intervals (CrIs) for the costs, DALYs and incremental results.

### Role of the funding source

The Brazilian National Malaria Programme and Medicines for Malaria Venture were involved in the design, analysis, writing, and the decision to submit for publication. Other funders were not involved in the study design, conduct, or analysis.

## Results

In the base case analysis, the *tafenoquine strategy* cost US$24 (R$122) more than *current practice* and US$13 (R$66) less than the *primaquine screening strategy* (Table 4). The *tafenoquine strategy* averted 0.008 more DALYs than both comparators. The ICER for the comparison with *current practice* was US$2894 (R$14,934), well below the willingness to pay threshold of US$7752 (R$40,000). Since the *tafenoquine strategy* averted DALYs while saving costs compared to the *primaquine screening strategy*, it dominated the *primaquine screening strategy* and an ICER was not calculated.

**Table 4:** Base case per person results for costs in 2020 United States dollars (US$) with Brazilian Real (R$) in parenthesis, disability-adjusted life-years (DALYs), and the incremental cost-effectiveness ratios (ICERs).

Strategy	Costs	Incremental cost	DALYs	DALYs averted	ICER
**First comparison**					
Current practice	US$15 (R$76)	base	0.022	base	base
Tafenoquine strategy	US$38 (R$198)	US$24 (R$122)	0.014	0.008	US$2894 (R$14,934)
**Second comparison**					
Primaquine screening strategy	US$51 (R$263)	base	0.022	Base	base
Tafenoquine strategy	US$38 (R$198)	−US$13 (−R$66)	0.014	0.008	dominates

**Fig. 1:**
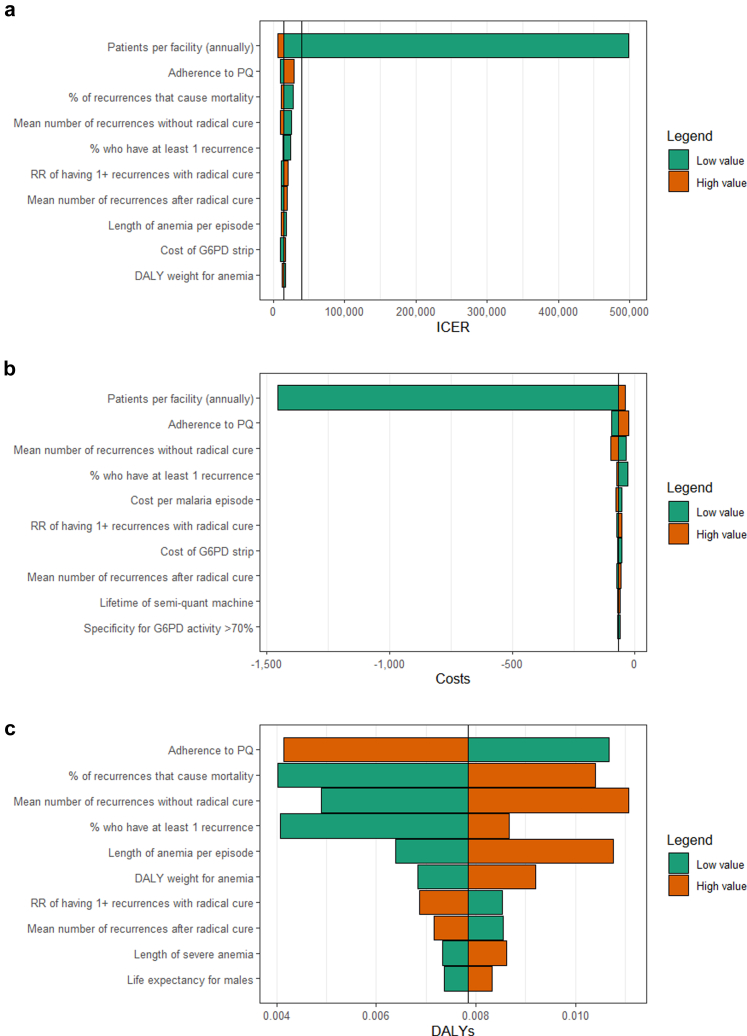
**Tornado diagrams for the ten most impactful parameters from the one-way sensitivity analyses.** Costs are in 2020 Brazilian Real (R$). a) Incremental cost-effectiveness ratio for *tafenoquine strategy* compared to *current practice* (an additional black line indicates the R$40,000 threshold). b) Incremental costs for *tafenoquine strategy* compared to *primaquine screening strategy*. c) DALYs averted for *tafenoquine strategy* compared to *primaquine screening strategy*. Low and high values for each parameter are listed in Table 1, Table 2, Table 3

The one-way sensitivity analysis was conducted on the ICER comparing the *tafenoquine strategy* with *current practice* (Fig. 1). Since the *tafenoquine strategy* dominated the *primaquine screening strategy*, the one-way sensitivity analysis was done on the costs and DALYs averted separately (Fig. 1). Across both comparisons, the number of patients per healthcare facility had the largest impact on the results, though this only impacted the costs. In the comparison with *current practice*, the ICER increased to US$96,899 (R$500,000) when the healthcare facility only treated one vivax malaria patient. For all other parameters, the one-way sensitivity analysis demonstrated that the ICER would remain below US$7752 (R$40,000), indicating that the *tafenoquine strategy* would remain very cost-effective. Other parameters that had a largest impact on the comparison of the *tafenoquine strategy* with *current practice* included adherence to primaquine, the proportion of *P. vivax* recurrences that cause mortality, the mean number of recurrences (both after radical cure and without radical cure), and the relative risk of having at least one recurrence after radical cure. These parameters also greatly impacted the comparison of the *tafenoquine strategy* with the *primaquine screening strategy*.

In the scenario analysis where 28% of males had intermediate G6PD deficiency, the ICER increased from US$2894 (R$14,934) to US$3769 (R$19,450) in the comparison between the *tafenoquine strategy* and *current practice* due to small increases in the incremental costs and decreases in the DALYs averted. When the discount rate was reduced to 0 to value future costs and benefits equivalently to the present, the comparison between the *tafenoquine strategy* and *current practice* ICER decreased to US$1388 (R$7161). When applying a discount rate of 10% to the analyser and DALYs, the ICER increased to US$3881 (R$20,024). When the above changes were applied to the *primaquine screening strategy*, it remained dominated by the *tafenoquine strategy*. The undiscounted results for comparing the *tafenoquine strategy* and the *primaquine screening strategy* were cost savings of US$12 (R$64) and 0.016 DALYs averted. When 10% discounting was applied to the results, the cost savings was US$13 ($R67) with 0.006 DALYs averted.

**Fig. 2:**
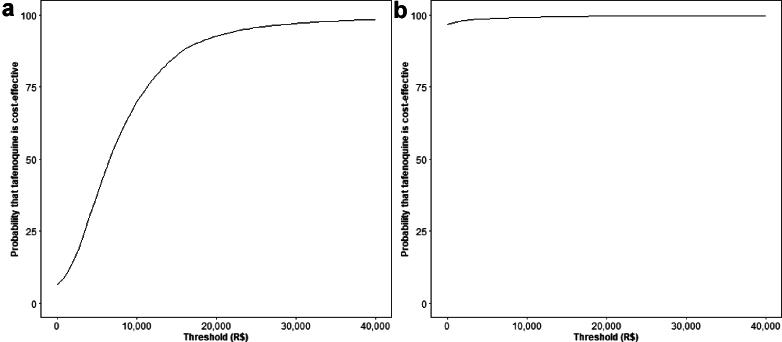
**Cost-effectiveness acceptability curves for *tafenoquine strategy* compared to a) *current practice* and b) *primaquine screening strategy* in 2020 Brazilian Real (R$)**.

The mean results of the PSA (Table 5) were similar to those of the base case (Table 4). For the scenarios that included semi-quantitative G6PD screening, the mean costs from the PSA were higher than those of the base case, reflecting the wide ranges used for the corresponding cost parameters. For the first comparison, the *tafenoquine strategy* consistently averted DALYs while the 95% CrI for the costs included values less than 0, indicating that it has the potential to be cost-saving. For the second comparison, the *tafenoquine strategy* saved costs (or had roughly equivalent costs to the *primaquine screening strategy*) and averted DALYs across all model iterations. The PSA showed that the tafenoquine strategy had 98.3% and 99.7% likelihoods of being cost-effective at a willingness-to-pay threshold of US$7752 (R$40,000) for the comparison with *current practice* and the *primaquine screening strategy*, respectively (Fig. 2). Since the *primaquine screening strategy* also had increased costs due to semi-quantitative G6PD screening, the tafenoquine strategy had a high likelihood of being cost-effective at a lower threshold than the comparison with *current practice*.

**Table 5:** Mean results including 95% credible intervals from probabilistic sensitivity analysis in 2020 United States dollars [Brazilian Real].

Strategy	Costs	Incremental costs	DALYs	DALYs averted	ICER
**First comparison**					
Current practice	US$15 (7, 25) [R$75 (37, 129)]	base	0.022 (0.011, 0.040)	base	base
Tafenoquine strategy	US$24 (4, 48) [R$125 (20, 250)]	US$10 (−11, 33) [R$ 50 (−57, 170)]	0.014 (0.007, 0.026)	0.008 (0.003, 0.016)	US$1534 (−1351, 6345) [R$ 7918 (−6971, 32,741)]
**Second comparison**					
Primaquine screening strategy	US$32 (4, 65) [R$165 (21, 335)	Base	0.022 (0.010, 0.040)	base	base
Tafenoquine strategy	US$24 (4, 48) [R$125 (20, 250)]	−US$8, (−21, 1) [−R$39 (−107, 4)]	0.014 (0.007, 0.026)	0.008 (0.003, 0.016)	dominates

## Discussion

This study evaluated the cost-effectiveness of tafenoquine prescription to vivax malaria patients aged 16 years or older who test G6PD normal compared to primaquine with and without G6PD screening in Brazil from the SUS perspective. The *tafenoquine strategy* demonstrated small but consistent health benefits in terms of DALYs in both comparisons. While the *tafenoquine strategy* increased costs by US$24 per person compared to primaquine without G6PD screening, it resulted in modest cost savings (US$13) compared to the *primaquine screening strategy*. The probabilistic sensitivity analysis showed that the *tafenoquine strategy* had a 98.3% likelihood of being cost-effective at a willingness-to-pay threshold of US$7752 in both comparisons. These results demonstrated that tafenoquine prescription to those who test G6PD normal with a semi-quantitative test will be cost-effective in Brazil. This was acknowledged through CONITEC's recent recommendation that tafenoquine following semi-quantitative G6PD screening be incorporated into the SUS, an essential milestone with Brazil becoming the first malaria-endemic country to introduce tafenoquine into its health system.

The cost-effectiveness of tafenoquine is attributable to reductions in hemolytic events and improvements in the effectiveness of radical cure through single-dose treatment. Compared to primaquine (low dose of 0.5 mg/kg for 7 days) after semi-quantitative G6PD screening, tafenoquine showed high certainty of saving costs while decreasing the number of years lost due to disability. This is due to improvements in the effectiveness of radical cure through single-dose treatment, while the cost of treatment with tafenoquine is not substantially higher than that of primaquine in Brazil. These findings may also be relevant for the treatment of relapsing *Plasmodium ovale*.

The one-way sensitivity analysis for the comparison between the *tafenoquine strategy* and *current practice* showed that the model results remained below the willingness to pay threshold of US$7752 for all parameter values except when the number of patients per facility per year was reduced from 55 to 1. This change increased the ICER to US$96,899. This is not surprising when one semi-quantitative G6PD analyser costs US$619. For healthcare facilities that see a minimal number of *P. vivax* patients, it is likely not to be cost-effective to screen for G6PD deficiency before treating with a radical cure. Conversely, when the number of patients per facility increased to 2385, the ICER reached its lowest value. This demonstrates how the cost per patient will be higher as areas move closer to malaria elimination and facilities see fewer vivax malaria patients. Accordingly, the short-term cost-effectiveness will decrease, which is generally true for malaria interventions in elimination settings. Since Brazil is committed to equitable access to treatment, all facilities that treat malaria patients must provide G6PD screening to enable tafenoquine prescription. Other parameters that had the most impact on the results included adherence to the primaquine regimen, parameters related to the risk and number of recurrences, and the risk of mortality for recurrences. These parameters have been shown to impact the results in previous cost-effectiveness studies significantly.^15^^,^^35^ While the application of these results to other settings may vary due to differences in costs and the epidemiology of vivax malaria and G6PD deficiency, the robustness of these findings should be reassuring, particularly where healthcare facilities expect to see a large number of patients annually.

Our study has several limitations, primarily regarding data availability to inform parameters. The risk and frequency of hemolytic events are poorly defined globally and make a large contribution to the model uncertainty due to their rarity. Fortunately, Brazil has some of the most extensive studies to inform these parameters.^11^ Brazil also has some of the best available data on the costs of hemolytic episodes and severe *P. vivax* treatment.^17^ As is often the case with new drugs and technologies, the costs for tafenoquine and the semi-quantitative test analyser, test strips, and quality assurance controls were not confirmed, so indicative prices from the test distributor and *Câmara de Regulação do Mercado de Medicamentos* (CMED) were used. Finally, a paediatric formulation was not available for those under the age of 16 at the time of the analysis. The paediatric formulation may be more expensive than the adult formulation, which could increase the ICER. Given that roughly two-thirds of vivax malaria patients are adults, this is unlikely to have a large impact.^36^

In conclusion, increased access to safe and effective radical cure will be critical to ensuring continued malaria control and accelerating the path to malaria elimination in Brazil. This comparison between single-dose tafenoquine after semi-quantitative G6PD screening and 7-day primaquine (with or without G6PD screening) indicates that tafenoquine is a cost-effective option for improving health outcomes for the population at risk of malaria in Brazil. Its implementation in Brazil will be a massive step forward for the safe and effective radical cure of vivax malaria locally while paving the way toward implementing tafenoquine throughout the Americas, particularly in regions exhibiting epidemiological and economic characteristics comparable to those observed in Brazil.

## Contributors

EJ, PGD, LLB and AD conceived and designed the initial proposal. HMP, JDBS, LLB, VS, PGD, EJ, WM, MVGL, AD contributed to data collection for the input parameters. AD acquired the funding, designed and analysed the model, visualised the results and wrote the original draft. All authors contributed to the interpretation of the results, verified the parameter values that were selected for the study, critically reviewed the article, and were responsible for the decision to submit the manuscript.

## Data sharing statement

All data generated and analysed in this study are openly accessible through the sources listed in Table 1, Table 2, Table 3 and Supplementary Table S1.

## Use of artifical intelligence

AI was not used in any aspect of the publication.

## Declaration of interests

PGD and EJ are employed by Medicines for Malaria Ventures.

## Appendix A Supplementary data

Supplementary Materials 2

Supp 1 - Resumo
