# Supplementary material for: The cost-effectiveness of tafenoquine following screening with STANDARD™ G6PD screening for the treatment of vivax malaria in the Brazilian Public Health System
Source: Lancet Reg Health Am. 2025 Sep 2;51:101216. doi: 10.1016/j.lana.2025.101216 (PMC12445694; doi:10.1016/j.lana.2025.101216)
Supplement: Supplementary Materials 2 [file mmc1.docx]

**SUPPLEMENTARY MATERIAL**

**The cost-effectiveness of tafenoquine following screening with STANDARD^TM^ G6PD screening for the treatment of vivax malaria in the Brazilian Public Health System**

**Table of contents**

[Figure S1. Diagram of decision tree model for males who have been diagnosed with vivax malaria for current practice and the tafenoquine strategy. 2](#_Toc188456292)

[Figure S2. Diagram of decision tree model for females who have been diagnosed with vivax malaria for current practice and the tafenoquine strategy. 3](#_Toc188456293)

[Supplementary Table 1. Radical cure given and outcomes of treatment in terms of recurrences by G6PD status for each strategy. 4](#_Toc188456294)

[CHEERS Checklist 5](#_Toc188456295)

Figure S1. Diagram of decision tree model for males who have been diagnosed with vivax malaria for current practice and the tafenoquine strategy. The *primaquine screening strategy* has the same structure as the *tafenoquine strategy* but primaquine is prescribed instead of tafenoquine. PQ = 7-day primaquine; PQ8W = 8 weekly doses of primaquine; TQ = tafenoquine.


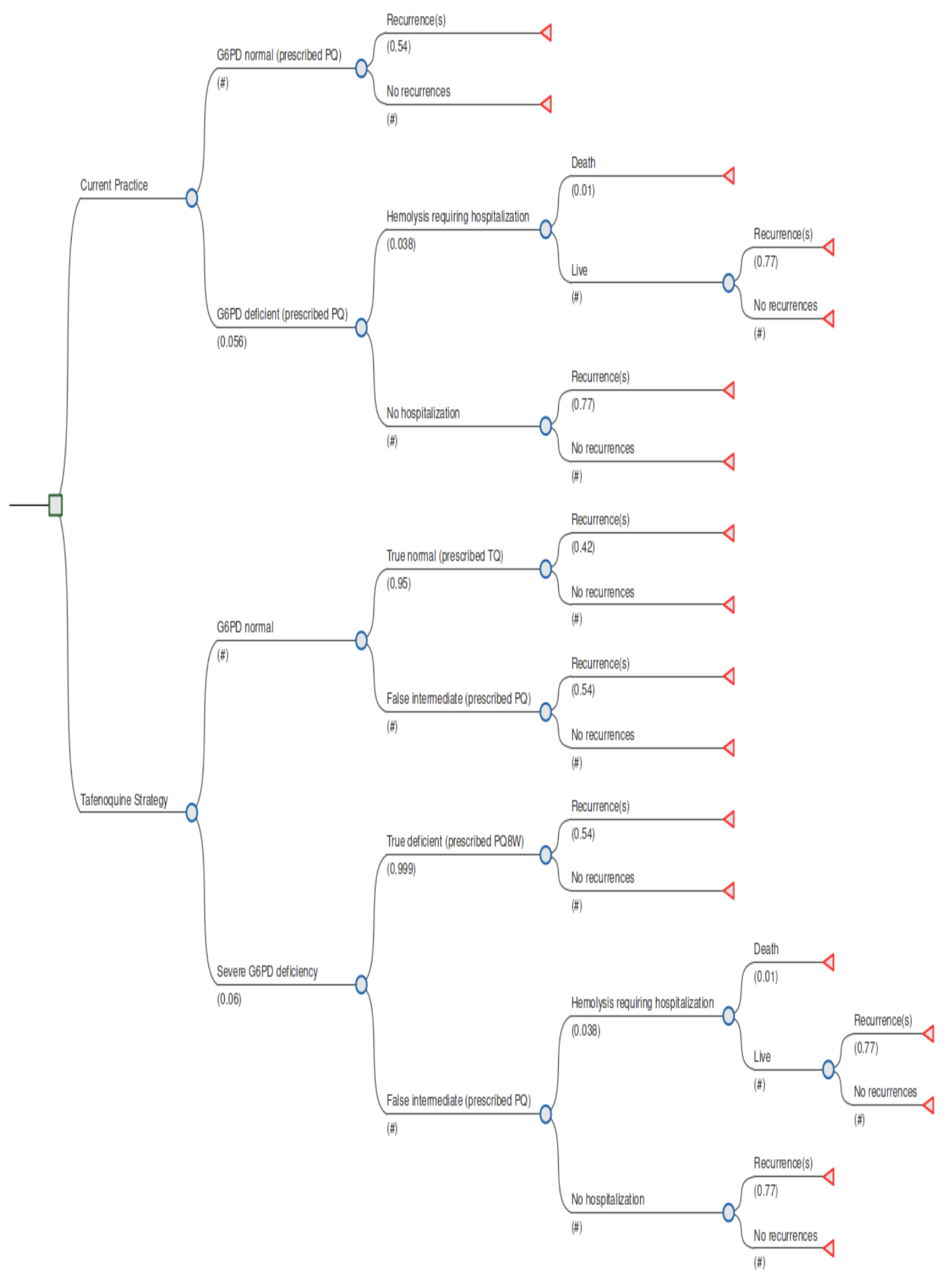


Figure S2 (next page). Diagram of decision tree model for females who have been diagnosed with vivax malaria for current practice and the tafenoquine strategy. The *primaquine screening strategy* has the same structure as the *tafenoquine strategy* but primaquine is prescribed instead of tafenoquine. PQ = 7-day primaquine; PQ8W = 8 weekly doses of primaquine; TQ = tafenoquine.


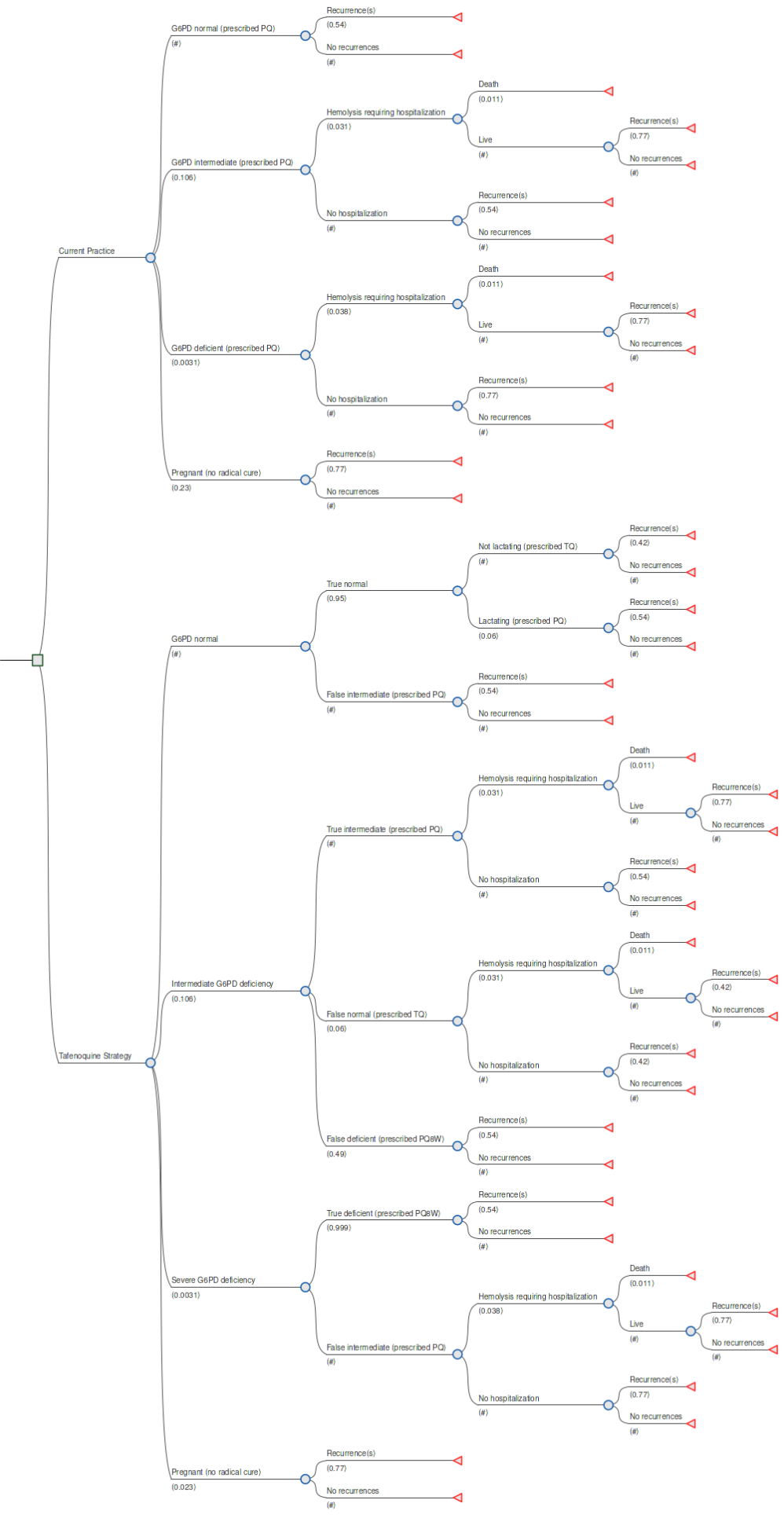


Supplementary Table 1. Radical cure given and outcomes of treatment in terms of recurrences by G6PD status for each strategy. Note that those receiving primaquine who had severe G6PD deficiency or had a severe hemolytic event requiring hospitalization with intermediate G6PD deficiency were assumed to not finish their primaquine course, so had the outcome of receiving chloroquine alone.

| **G6PD status** | **Test result** | **Severe hemolysis requiring hospitalization** | **Current practice** | **Primaquine screening strategy** | **Tafenoquine strategy** |
| --- | --- | --- | --- | --- | --- |
| Severe G6PD deficiency (<30% activity) | True abnormal | Yes | CQ | CQ | MR^a^ |
|  |  | No |  |  |  |
|  | False intermediate | Yes |  | CQ | CQ |
|  |  | No |  |  |  |
| Intermediate G6PD deficiency (30-70% activity) | Correctly classified as 30-70% activity | Yes | CQ | CQ | CQ |
|  |  | No | MR | MR | MR |
|  | Incorrectly classified as G6PD normal | Yes | CQ | CQ | TQ |
|  |  | No | MR | MR | TQ |
|  | Incorrectly classified as severe G6PD deficiency | Yes | CQ | MR | MR |
|  |  | No | MR |  |  |
| G6PD normal (>70% activity) | True normal | N/A | MR | MR | TQ |
|  | False abnormal | N/A |  | MR | MR |

Abbreviations: CQ-effectiveness of chloroquine, TQ-effectiveness of tafenoquine, MR-mixed result of CQ and primaquine weighted by adherence.

^a^Where $MR =propR*RR* {NR}_{rc}*propA+ propR*{NR}_{cq}*\left( 1-propA \right)$. $propR$ is the proportion expected to have at least 1 recurrence, $RR$ is the relative risk of having a recurrence if not prescribed radical cure, ${NR}_{rc}$ is the expected relapses with radical cure, ${NR}_{cq}$ is the expected relapses without radical cure and $propA$ is the proportion adherent to 7-day or 8-weekly primaquine.

# CHEERS Checklist

| **Topic** | **No.** | **Item** | **Location where item is reported** |
| --- | --- | --- | --- |
| **Title** |  |  |  |
|  | 1 | Identify the study as an economic evaluation and specify the interventions being compared. | page 1 |
| **Abstract** |  |  |  |
|  | 2 | Provide a structured summary that highlights context, key methods, results, and alternative analyses. | page 2 |
| **Introduction** |  |  |  |
| **Background and objectives** | 3 | Give the context for the study, the study question, and its practical relevance for decision making in policy or practice. | pages 6-7 |
| **Methods** |  |  |  |
| **Health economic analysis plan** | 4 | Indicate whether a health economic analysis plan was developed and where available. | Not reported |
| **Study population** | 5 | Describe characteristics of the study population (such as age range, demographics, socioeconomic, or clinical characteristics). | pages 7-8 |
| **Setting and location** | 6 | Provide relevant contextual information that may influence findings. | page 7 |
| **Comparators** | 7 | Describe the interventions or strategies being compared and why chosen. | page 8 |
| **Perspective** | 8 | State the perspective(s) adopted by the study and why chosen. | page 7 |
| **Time horizon** | 9 | State the time horizon for the study and why appropriate. | page 7 |
| **Discount rate** | 10 | Report the discount rate(s) and reason chosen. | page 13 |
| **Selection of outcomes** | 11 | Describe what outcomes were used as the measure(s) of benefit(s) and harm(s). | page 11 |
| **Measurement of outcomes** | 12 | Describe how outcomes used to capture benefit(s) and harm(s) were measured. | pages 10-11 |
| **Valuation of outcomes** | 13 | Describe the population and methods used to measure and value outcomes. | page 11 |
| **Measurement and valuation of resources and costs** | 14 | Describe how costs were valued. | pages 10-11 |
| **Currency, price date, and conversion** | 15 | Report the dates of the estimated resource quantities and unit costs, plus the currency and year of conversion. | page 10 |
| **Rationale and description of model** | 16 | If modelling is used, describe in detail and why used. Report if the model is publicly available and where it can be accessed. | pages 7-10 |
| **Analytics and assumptions** | 17 | Describe any methods for analysing or statistically transforming data, any extrapolation methods, and approaches for validating any model used. | Not applicable |
| **Characterising heterogeneity** | 18 | Describe any methods used for estimating how the results of the study vary for subgroups. | pages 7-8 |
| **Characterising distributional effects** | 19 | Describe how impacts are distributed across different individuals or adjustments made to reflect priority populations. | pages 7-8 |
| **Characterising uncertainty** | 20 | Describe methods to characterise any sources of uncertainty in the analysis. | pages 11-12 |
| **Approach to engagement with patients and others affected by the study** | 21 | Describe any approaches to engage patients or service recipients, the general public, communities, or stakeholders (such as clinicians or payers) in the design of the study. | page 12 |
| **Results** |  |  |  |
| **Study parameters** | 22 | Report all analytic inputs (such as values, ranges, references) including uncertainty or distributional assumptions. | Tables 1-3 |
| **Summary of main results** | 23 | Report the mean values for the main categories of costs and outcomes of interest and summarise them in the most appropriate overall measure. | page 12 |
| **Effect of uncertainty** | 24 | Describe how uncertainty about analytic judgments, inputs, or projections affect findings. Report the effect of choice of discount rate and time horizon, if applicable. | pages 12-14 |
| **Effect of engagement with patients and others affected by the study** | 25 | Report on any difference patient/service recipient, general public, community, or stakeholder involvement made to the approach or findings of the study | Not reported |
| **Discussion** |  |  |  |
| **Study findings, limitations, generalisability, and current knowledge** | 26 | Report key findings, limitations, ethical or equity considerations not captured, and how these could affect patients, policy, or practice. | pages 14-16 |
| **Other relevant information** |  |  |  |
| **Source of funding** | 27 | Describe how the study was funded and any role of the funder in the identification, design, conduct, and reporting of the analysis | page 17 |
| **Conflicts of interest** | 28 | Report authors conflicts of interest according to journal or International Committee of Medical Journal Editors requirements. | page 17 |

*From:* Husereau D, Drummond M, Augustovski F, et al. Consolidated Health Economic Evaluation Reporting Standards 2022 (CHEERS 2022) Explanation and Elaboration: A Report of the ISPOR CHEERS II Good Practices Task Force. Value Health 2022;25. <doi:10.1016/j.jval.2021.10.008>
