# Supplementary material for: The cost-effectiveness of tafenoquine following screening with STANDARD™ G6PD screening for the treatment of vivax malaria in the Brazilian Public Health System
Source: Lancet Reg Health Am. 2025 Sep 2;51:101216. doi: 10.1016/j.lana.2025.101216 (PMC12445694; doi:10.1016/j.lana.2025.101216)
Supplement: Supp 1 - Resumo [file mmc2.docx]

**CONTEXTO**
A malária por *Plasmodium vivax* requer cura radical para eliminar tanto os parasitas da fase sanguínea quanto os da fase hepática. O Brasil, como a maioria dos países endêmicos, tem prescrito primaquina em regime de 7 dias para a cura radical, sem realizar testes para deficiência de glicose-6-fosfato desidrogenase (G6PD), o que impede a exclusão de indivíduos com risco de hemólise induzida pela primaquina. A tafenoquina, um novo medicamento de dose única para cura radical, exige triagem de G6PD antes da prescrição, a fim de garantir segurança. Este estudo tem como objetivo avaliar a relação custo-efetividade da prescrição da tafenoquina após triagem semi-quantitativa de G6PD, na perspectiva do Sistema Único de Saúde (SUS) brasileiro.

**MÉTODOS**
Foi desenvolvido um modelo de árvore de decisão para adultos com malária vivax ao longo de 12 meses. A estratégia com tafenoquina — triagem semi-quantitativa de G6PD antes da prescrição da dose única de tafenoquina para indivíduos com ≥ 70% de atividade enzimática — foi comparada com: (1) prática atual: primaquina em baixa dose por 7 dias (0,5 mg/kg/dia), sem triagem de G6PD; e (2) estratégia com triagem para primaquina: mesma dose de primaquina por 7 dias para pacientes com ≥ 30% de atividade enzimática, conforme teste semi-quantitativo. O desfecho principal foi o custo por ano de vida ajustado por incapacidade (DALY) evitado, em comparação com o limiar de disposição a pagar no Brasil de US$7.752 (R$40.000).

**RESULTADOS**
A estratégia com tafenoquina custou US$2.894 (R$14.934) por DALY evitado, em comparação com a prática atual, ficando bem abaixo do limiar de custo-efetividade. Comparada à estratégia com triagem para primaquina, a estratégia com tafenoquina evitou 0,14 DALYs e gerou uma economia de US$13 (R$66). Em ambas as comparações, a estratégia com tafenoquina apresentou mais de 98% de probabilidade de ser custo-efetiva.

**INTERPRETAÇÃO**
A prescrição de tafenoquina para indivíduos com resultado normal no teste semi-quantitativo de G6PD é uma estratégia custo-efetiva para a cura radical da malária *vivax* no Brasil. Embora a custo-efetividade possa variar em outros contextos, devido a diferenças de custos e à epidemiologia da malária *vivax* e da deficiência de G6PD, a robustez desses achados é encorajadora, especialmente em locais onde os serviços de saúde atendem grande volume de pacientes anualmente.
